# Adjuvant FOLFOX treatment for stage III colon cancer: how many cycles are enough?

**DOI:** 10.1186/s40064-016-2976-9

**Published:** 2016-08-11

**Authors:** Yi-Jian Tsai, Jen-Kou Lin, Wei-Shone Chen, Jeng-Kai Jiang, Hao-Wei Teng, Chueh-Chuan Yen, Tzu-chen Lin, Shung-Haur Yang

**Affiliations:** 1 Division of Colon & Rectal Surgery, Department of Surgery, Taipei Veterans General Hospital, No 201,Sec 2, Shih-Pai Rd, Taipei, 11217 Taiwan; 2 School of Medicine, National Yang-Ming University, Taipei, Taiwan; 3 Division of Medical Oncology, Department of Oncology, Taipei Veterans General Hospital, Taipei, Taiwan

**Keywords:** Adjuvant chemotherapy, FOLFOX, Colon cancer, Oxaliplatin

## Abstract

**Purpose:**

Adjuvant FOLFOX (5-fluorouracil and oxaliplatin) chemotherapy benefits stage III colon cancer patients. However, it still results in side effects and increased cost. Reducing cycles had been thought to decrease these problems. This retrospective study aimed to find the appropriate number of treatment cycles that are sufficient for treating these patients.

**Patients and methods:**

A total of 213 stage III colon cancer patients receiving adjuvant FOLFOX therapy were retrospectively recruited. Demographic data were collected for analysis. Survival analyses were performed between all cases of patients receiving above and below a certain cycle number. If a significant difference was reached at that cycle number, multivariate Cox Regression was performed with those factors resulting in p < 0.2 to assess the independent prognostic factors.

**Results:**

The 5-year overall survival rate of patients was 77.9 %, and the 3-year disease-free survival was 76.7 %. For overall survival, a significant benefit was noted for treatment of at least 8 cycles, for disease-free survival, significant differences were apparent from patient data of those who underwent from 7 to 12 treatment cycles. Multivariate survival analysis of that patient data at cycle 8 for overall survival and cycle 7 for disease free survival revealed cycle number as the only independent prognostic factor (p = 0.04, 0.048).

**Conclusion:**

Cycle number of adjuvant FOLFOX is a significant prognostic factor for stage III colon cancer patients. At least 8 cycles are needed to have an overall survival benefit, and 7 to disease-free survival.

## Background

For patients with colon cancer, surgical resection offers the only potential cure. Post-operative adjuvant chemotherapy has been demonstrated to improve outcomes in high-risk patients since the 1990s, and it has become standard treatment (O’Connell et al. 1997; Moertel et al. 1990; Wolmark et al. 1993). The results of the Multicenter International Study of oxaliplatin/5-fluorouracil/leucovorin in the adjuvant treatment of colon cancer (MOSAIC) trial in 2004 (Andre et al. 2004), along with the National Surgical Adjuvant Breast and Bowel Project (NSABP) C-07 report in 2007 (Kuebler et al. 2007), revealed that adding oxaliplatin to a regimen of fluorouracil (FU) combined with leucovorin (LV) produced a significant improvement in 3-year disease-free survival (DFS). FOLFOX increased overall survival in the MOSAIC trial (Andre et al. 2009). After these findings, 12 cycles of FOLFOX [folinic acid (leucovorin), fluorouracil, and oxaliplatin] became the standard adjuvant regimen for stage III colon cancer treatment.

Despite the efficacy of FOLFOX treatment for stage III colon cancer, this treatment leads to significant cost increase, toxicity, and inconvenience. In particular, oxaliplatin-induced cumulative dose-dependent neurotoxicity is a clinically relevant issue. Peripheral neuropathy was reported for 92.1 % of patients receiving treatments, and the incidence of grade 3 neurotoxicity 1 year after completion was estimated to be 12 % in the MOSAIC trial. Approximately 50 % of patients suffered from grade 1 or 2 neurotoxicity in the second post-treatment year (Andre et al. 2004, 2009).

Previous studies showed that oxaliplatin-induced neurotoxicity presented at the cumulative dose of 785–850 mg/m^2^, (de Gramont et al. 2000; Grothey et al. 2000; Grothey 2005) which corresponds to the 8–10th cycle of FOLFOX. We wanted to examine if FOLFOX cycles could be reduced in order to lessen the associated neurotoxicity, while preserving the survival effects of treatment. No trial has compared different numbers of FOLFOX cycles yet. However, there were some findings regarding oxaliplatin dosage in the MOSAIC and NSABP C-07 studies (Andre et al. 2009; Kuebler et al. 2007). In the MOSAIC trial, the median oxaliplatin dose received per patient in the FOLFOX4 group was 810 mg/m^2^, 79.4 % of the 12-cycle protocol dose (1020 mg/m^2^). In the FLOX group of the NSABP C-07 trial, the median oxaliplatin dose was 677 mg/m^2^, 88.5 % of the 9-cycle protocol dose (765 mg/m^2^). The median dose of the NSAPBP C-07 FLOX group was 66.4 % of the 12-cycle dosage.

If a reduced number of FOLFOX cycles can have the same survival benefit and decreased side effects, it would be beneficial to both patients and care providers. It is our aim, through a retrospective review, to evaluate the number of adjuvant FOLFOX cycles that are necessary to achieve survival benefits for stage III colon cancer patients.

## Results

A total of 692 consecutive cases of stage III colorectal cancer were collected in the section of colorectal surgery database at Taipei Veterans General Hospital. Among them, 210 cases were excluded due to inadequate data or lack of follow-up, 49 were excluded for not receiving any chemotherapy, and 220 were excluded for receiving chemotherapy other than FOLFOX. As a result, 213 cases of patients with stage III colon adenocarcinoma treated with adjuvant mFOLFOX6 were analyzed. Table 1 shows demographic data. Among the study population of median age 61.7 years-old, there was a predominant distribution of males (54.5 %), low preoperative CEA (<5 ng/mL; 61.5 %), pT1–3 (90.6 %), pN1 (60.1 %), and left side colon localization (59.6 %). There were also high rates of well- or moderately-differentiated tumors (88.3 %), patients receiving adjuvant chemotherapy within 8 weeks of the operation (95.3 %), patients who had more than 12 lymph nodes harvested (91.1 %), and patients with an ECOG performance status grade of 0 (90.6 %).

**Table 1:** Demographic data of patients

Sex		
Men	114	54.50 %
Woman	99	45.50 %
Age (years)		
Median (range)	61.65	29–88
<70	152	71.40 %
≥70	61	28.60 %
Performance status		
0	193	90.60 %
1	17	8.00 %
2	3	1.40 %
Median OPD follow-up (months)	54.03	
5 year survival rate	77.90 %	
5 year cancer specific survival	82.20 %	
3 year disease free survival	76.70 %	
Recurrence rate	26.30 %	
Pathological staging		
pT classification		
pT1–3	193	90.60 %
pT4	20	9.40 %
pN classificaton		
pN1	128	60.10 %
pN2	85	39.90 %
Tumor localization		
Right	85	40.40 %
Left	128	59.60 %
Obstruction/Perforation	35	16.40 %
Histopathology grade		
Well, and moderate differentiated	188	88.30 %
Poor differentiated	22	10.30 %
Missing	3	1.40 %
Pre-operative CEA level		
CEA >5	70	32.9 %
CEA ≤5	131	61.5 %
Missing	12	5.6 %
Time to chemotherapy (weeks)		
≥8	10	4.70 %
<8	203	95.30 %
Lymph nodes harvested		
Lymph nodes <12	19	8.90 %
Lymph nodes ≥12	194	91.10 %

The median follow-up period was 54.0 months. The recurrence rate was 26.3 %. The 5-year OS was 77.9 %, and the 3-year DFS was 76.7 %.

Table 2 shows the cumulative percentage of patients fulfilling each cycle number of chemotherapy. One hundred and eighteen patients (55.4 %) completed the full 12 cycles. Of the 95 patients (44.6 %) not completing full cycles (CFC), 81 patients (85.3 %) received 5-FU based chemotherapy as replacement chemotherapy, via either oral or intravenous administration.

**Table 2:** Patients complete each FOLFOX cycle

FOLFOX cycles		(%)	Cumulative percentage (%)
Total	213		
1	9	4.2	4.2
2	5	2.3	6.6
3	6	2.8	9.4
4	8	3.8	13.1
5	5	2.3	15.5
6	29	13.6	29.1
7	5	2.3	31.5
8	9	4.2	35.7
9	7	3.3	39.0
10	10	4.7	43.7
11	2	0.9	44.6
12	118	55.4	100.0

**Fig. 1:**
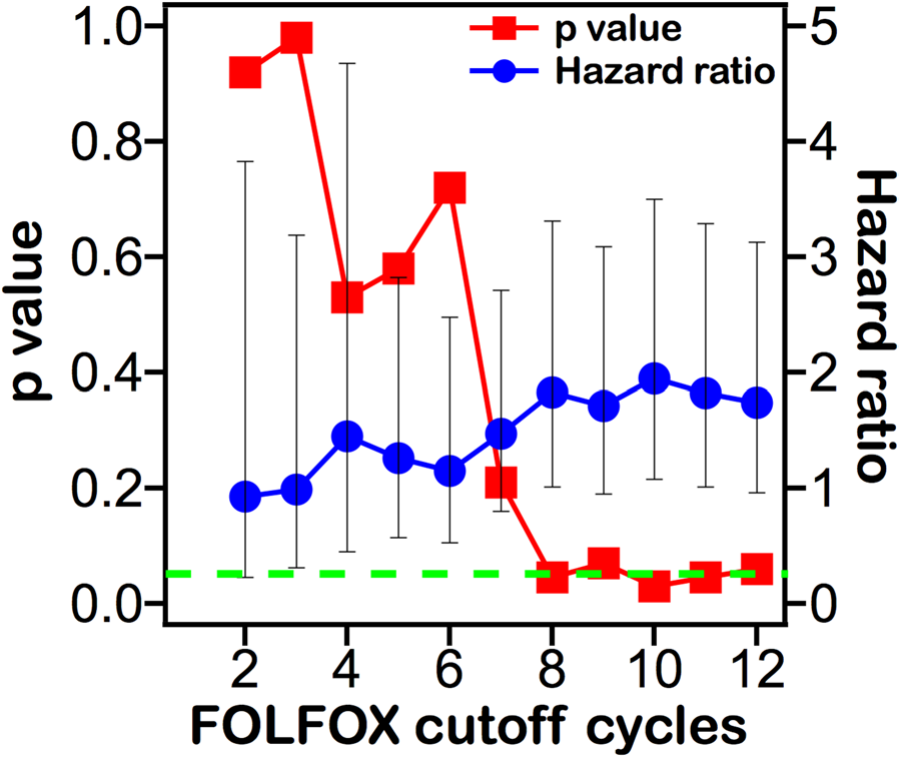
p value and hazard ratio with 95 % confidence interval of patients stratified to each cycle of treatment for overall survival

For overall survival (OS) of colorectal cancer patients (Fig. 1), the trend is apparent. A significant difference was noted when comparing groups of patients that received at least 8 cycles and those that received less than 8 cycles. When differentiating between groups using conditions of more than 8 cycles, the p value decreased dramatically and fluctuated around 0.05, with use of 9 cycles resulting in p = 0.07 and use of 12 cycles resulting in p = 0.06. Multivariate survival analysis of patients stratified according to 8 cycles of treatment (Table 3), also including pT, ECOG, lymph node number, and perforation/obstruction, revealed treatment cycle number as the only independent prognostic factor (p = 0.04).

**Table 3:** Multivariate survival analysis of patients stratified according to 8 cycles of treatment for overall survival

	Univariate COX regression			Multivariats logistic regression		
	Hazard ratio	95 % Cl	p value	Hazard ratio	95 % Cl	p value
Sex (man vs woman)	1.13	0.62–2.04	0.7			
Age (≥70 years vs <70 years)	1.22	0.65–2.23	0.53			
T stage (4 vs 1–3)	1.77	0.79–3.97	0.17	1.21	0.51–2.89	0.67
N stage (2 vs 1)	1.31	0.73–2.37	0.37			
Histo-pathological grade (poor differentiated vs. well, moderated differentiated)	1.51	0.78 –5.23	0.35			
Site (right vs left)	1.1	0.61–1.98	0.76			
Pre-operative CEA (≥ 5 vs. < 5)	0.9	0.46–1.75	0.76			
Performance status (1 and 2 vs.0)	2.08	0.93–4.66	0.08	1.91	0.83–4.40	0.13
FOLFOX cycles (≥8 vs <8)	0.54	0.30–0.99	0.043	0.52	0.28–0.97	0.038
Lymph nodes (≥12 vs. <12)	3.1	0.75–12.89	0.12	2.9	0.68–12.31	0.15
Time to treatment (≥8 vs <8 weeks)	1.71	0.61–4.79	0.31			
Perforation or obstruction	1.92	0.99–3.73	0.053	1.41	0.68–2.93	0.36

**Fig. 2:**
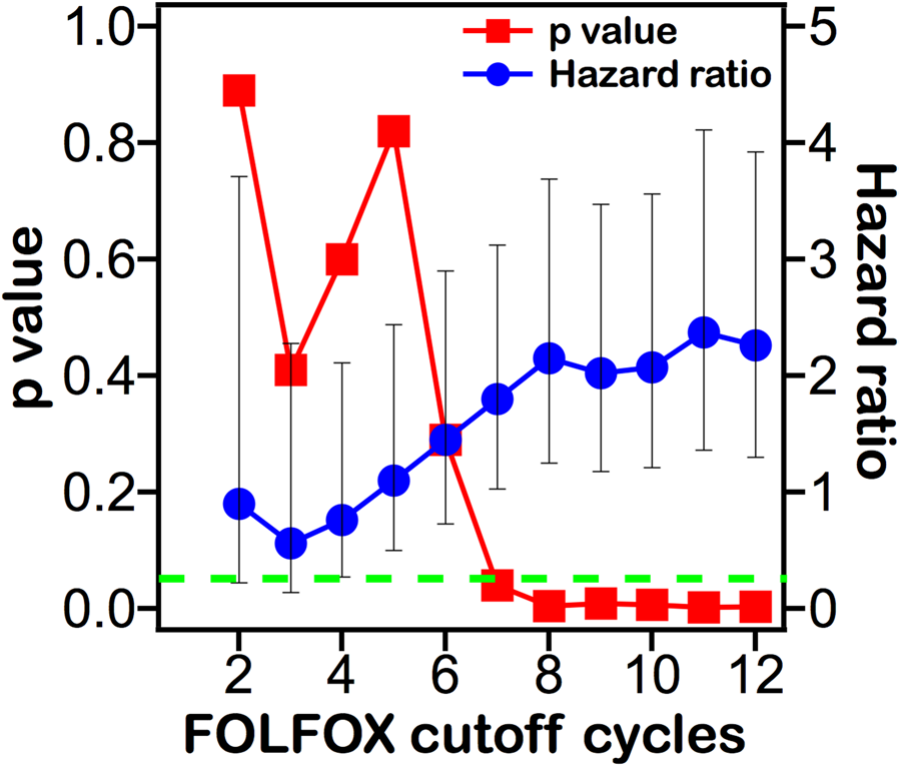
p value and hazard ratio with 95 % confidence interval of patients stratified to each cycle of treatment for disease free survival

For disease free survival (DFS) (Fig. 2), significant difference was noted when comparing groups of patients that received at least 7 cycles and those that received less than 7 cycles, significance was maintained for increasing numbers of cycles up to 12. Multivariate survival analysis of patients stratified according to 7 cycles of treatment (Table 4), also including time to chemotherapy, revealed cycle number as the only independent prognostic factor (p = 0.048).

**Table 4:** Multivariate survival analysis of patients stratified according to 7 cycles of treatment for disease-free survival

	Univariate COX regression			Multivariats logistic regression		
	Hazard ratio	95 % Cl	p value	Hazard ratio	95 % Cl	p value
Sex (man vs woman)	0.94	0.55–1.62	0.83			
Age (≥70 years vs <70 years)	0.9	0.49–1.65	0.73			
T stage (4 vs 1–3)	1.63	0.70–3.81	0.26			
N stage (2 vs 1)	1.01	0.59–1.76	0.96			
Histo-pathological grade (poor differentiated vs. well, moderated differentiated)	0.73	0.262–2.017	0.539			
Site (right vs left)	1.08	0.62–1.86	0.8			
Pre-operative CEA (≥5 vs. <5)	0.79	0.42–1.47	0.45			
Performance status (1 and 2 vs.0)	1.07	0.43– 0.68	0.89			
FOLFOX cycles (≥7 vs. <7)	0.56	0.32–0.97	0.04	0.57	0.33–0.996	0.048
Lymph nodes (≥12 vs. <12)	0.67	0.300–1.48	0.32			
Time to treatment (≥8 vs <8 weeks)	2.22	0.08–6.15	0.12	2.2	0.73–5.64	0.18
Perforation or obstruction	1.52	0.78–2.96	0.21			

## Discussion

Cycle number does matter. This study reveals that survival benefits of adjuvant FOLFOX treatment for stage III colon cancer patients require at least 7 or 8 cycles. Specifically, 8 cycles are required for OS benefits, and 7 cycles are required for DFS benefits. It is very likely that those OS and DFS benefits are also conferred to patients who receive up to 12 cycles.

The initial demonstration of efficacy of adjuvant 5-FU plus levamisole treatment was based on a study of 12-month treatment of colon cancer patients (Moertel et al. 1990). Subsequent studies confirmed that 6-month treatment resulted in similar effects (Haller et al. 2005; Andre et al. 2003, 2007), and 3 months of protracted venous 5-FU infusion was shown to be as effective as 6 months of standard bolus 5-FU/leucovorin and significantly less toxic (Chau et al. 2005). As for FOLFOX, our results suggest that treatments should be given in as many courses as possible up to the complete course, and if that is not possible, at least 7 cycles should be administered. However, as stated earlier, the annoying neurotoxicity side effect of FOLFOX appears at the 8–10th cycle administration. This time period is the critical time to gain or lose survival benefits. Although treatment series of fewer cycles showed some potential to ameliorate this neurotoxicity (Hosokawa et al. 2012; Milla et al. 2009), recent studies failed to show any convincing benefit (Oki et al. 2015; Pachman et al. 2015; Zimmerman et al. 2015; Loprinzi et al. 2014), even on a molecular basis (Ruzzo et al. 2014). It is still a challenge to be solved. Any “wait and go” policy to reduce side effects needs to be evaluated in a larger cohort of patients (Kochi et al. 2011). Our results notice the importance of treatments reducing the likelihood of adverse effect. This should be verified on solid base.

Patient CFC rate for this study was 55.9 %, which is lower than the 74.7 % in the MOSAIC trial. However, the 6-year OS rate (72.6 %) and 3-year DFS (75.6 %) of our patients are close to of the data from the MOSAIC study (72.9 and 78.2 %, respectively). In the NSABP C-07 trial, the 3-year DFS was 76.1 % for FLOX arm (Andre et al. 2004; Kuebler et al. 2007). However, the two studies both included stage II colon cancer patients in whom benefit of adjuvant chemotherapy is small and the survival outcome was more favorable. This CFC rate for stage III patients is similar to that of another cohort of American veteran stage III colorectal cancer patients (58 %) (Aspinall et al. 2015). Over 90 % of patients after oxaliplatin disruption had maintenance treatments. The maintenance treatment cycles were not included in total accumulative cycles. This might be the reason of our good outcome.

We did collect the reasons for discontinuing oxaliplatin. Half (50.5 %) were due to intolerance of adverse effect. Physicians’ will to avoid adverse effects in advance accounts for the other 23.2 %. Disease progression made the switching to other regimen explained another 16.8. The remaining 9.5 % was patients’ will to terminate without apparent reasons.

This retrospective and small population study had many shortcomings. These comparisons at certain cutoff cycle numbers are comparisons of heterogeneous populations: all patients who received below a certain number of cycles compared to those that received above that number. It is not a head-to-head comparison between two different cycle numbers. This was because we had a small patient population satisfying the inclusion criteria. Furthermore, cumulative dose intensity was not included here. We agree it would strengthen our study if it was analyzed. Recently, Kumar et al. (2015) published a report in which they used a similar analysis method during a similar period and showed that early discontinuation of FOLFOX (cycle of FOLFOX, ≥10 vs <10) was not associated with differences in survival outcomes. They also mentioned similar findings after titrating cycle number at 9, and 11. Their survival benefit persisted under considering the number of treatment cycles as a continuous variable when examined for survival. We do not have a good explanation for this contrary finding. Reviewing the demographic distribution, we found that the major difference in patient data was that they had a population 2 times bigger than ours. However, there was no information regarding the ratio fulfilling each cycle. A higher ratio of our patients received chemotherapy within 8 weeks after operations.

Additional, with a CFC rate of 55.4 %, the comparison would be more weighted toward patients receiving a larger number of FOLFOX cycles. This may be the main reason for persistent significant differences up to the last cycle. The other major possible contributing factor is that 85.6 % of those who did not receive CFC received other 5-FU based replacements that were administered orally or intravenously to finish the scheduled course. This was not included in analysis. All of these potential issues are problems that can be associated with a retrospective study.

Currently, the International Duration Evaluation of Adjuvant chemotherapy (IDEA) collaboration is undertaking a prospective analysis combining several randomized trials of FOLFOX (6 vs. 12 cycles) or Capecitabine plus Oxaliplatin(XELOX) (4 vs. 8 cycles) for stage III colon cancer treatment (Andre et al. 2013). Researchers want to evaluate if half of the standard cycle number is non-inferior to a full complement of treatment cycles with respect to 3-year DFS. However, our results reveal that at least 7 cycles may be needed to obtain the DFS benefit.

In conclusion, the number of adjuvant FOLFOX cycles administered matters in stage III colon cancer therapy. At least 8 cycles are needed to increase OS, and 7 cycles are necessary to prolong DFS. The DFS benefits are also present for patients received more cycles of chemotherapy up to the full course.

## Methods

### Study setting and population

This is a retrospective observation cohort study of patients treated between June 2005 and June 2012. Cases were retrieved from the section of colorectal surgery database at Taipei Veterans General Hospital. Cases of stage III colon adenocarcinoma in which patients underwent curative resections and adjuvant FOLFOX chemotherapy were retrieved. The primary colon tumor site was defined as the region from the cecum to the sigmoid colon, excluding the rectum and recto-sigmoid junction. No patients received neoadjuvant chemotherapy or radiotherapy.

Patients were followed through June 2015. The standard follow-up schedule was set at 3-month intervals for up to 2 years, 6-month intervals for up to 5 years, and then annually. During follow-up visits, all relevant data were collected. Patient with lost follow-up was excluded.

The Eastern Cooperative Oncology Group (ECOG) method was used to quantify general performance status (Sargent et al. 2005).

All patients signed informed consents to be enrolled into the database. This study was approved by the Local Survey and Behavior Ethics Committee.

### Chemotherapy regimens

A modified FOLFOX6 (mFOLFOX6) regimen was adopted. Oxaliplatin was administered via 2-h intravenous infusion (IVI) at a dose of 85 mg/m^2^. Then, Leucovorin was administered via 2-h IVI at a dose of 400 mg/m^2^, followed by a 5-FU bolus at a dose of 400 mg/m^2^ on day 1, and then 5-FU was administered via 46 to 48-h IVI a dose of 2400 mg/m^2^ from day 1. The next cycle was scheduled for 2 weeks later.

### Primary outcome measures

The primary outcomes were overall survival (OS) and disease-free survival (DFS). OS was defined as the time from operation day to death from any cause. DFS was defined as the time from operation day to either colon cancer recurrence or death, whichever occurred first. Previously, 3-year DFS has been used as a surrogate marker for OS in clinical trials of adjuvant chemotherapy for colon cancer (Sargent et al. 2005).

### Statistical analysis

General clinicopathological data were collected for analyses, including age, sex, preoperative carcinoembryonic antigen (CEA), detailed pT and pN staging, tumor site, obstruction/perforation, differentiation, lymph node number (set at 12), and ECOG performance status, and if to receive chemotherapy within 8 weeks. The Kaplan–Meier technique (primary analysis) and compared survival with a stratified two-sided log-rank test were used to determine statistical significance. Cases were stratified by the number of FOLFOX cycles that patients received. In the order of titrating cycle numbers increasingly, survival analyses were done between all those cases above a certain cycle number (included) and all cases below that cycle number. If a significant difference was reached at certain cycle number, univariate Cox Regression was done for every demographic factor, including the number of cycles administered. Multivariate cox regression was further performed with all those factors resulting in p < 0.2 to determine if that cycle number was an independent prognostic factor. Statistical analyses were performed using SPSS, version 22 (IBM Corp. Armonk, New York, USA). Significance was set at p < 0.05.
